# Recovery of the physiological status in professional basketball players using NESA neuromodulation treatment during different types of microcycles in season: A preliminary randomized clinical trial

**DOI:** 10.3389/fphys.2022.1032020

**Published:** 2022-11-22

**Authors:** F. García, D. Fernández, J. Vázquez-Guerrero, R. Font, B. Moreno-Planas, D. Álamo-Arce, R. Medina-Ramírez, M. Mallol-Soler

**Affiliations:** ^1^ Sports Performance Area, Futbol Club Barcelona, Barcelona, Spain; ^2^ Barça Innovation Hub, Futbol Club Barcelona, Barcelona, Spain; ^3^ Physical Therapy, University Francisco de Vitoria, Pozuelo de Alarcón, Spain; ^4^ SocDig Research Group, University of Las Palmas de Gran Canaria, Las Palmas, Spain

**Keywords:** recovery, basketball, competition, biomarkers, neuromodulation

## Abstract

The purpose of the study was to describe and compare recovery status after official basketball competition in players who underwent NESA neuromodulation treatment (NNT) in weeks with one or two matches. The recovery parameters of 12 professional male basketball players (mean ± SD, age: 20.6 ± 2.7 yr; height: 197.8 ± 11.7 cm; and body mass: 89.0 ± 21.2 kg) that competed in the LEB Plata (Spanish third division) were monitored 2 days after match-play over 6 weeks, and included: 1) the Hooper Test, which combines four subjective variables (sleep, stress, fatigue and soreness); 2) common biochemical markers (e.g., testosterone, cortisol and ratio T:C); and 3) lowest heart rate [HR], average HR, HR variability, sleep duration, awake time during night and onset latency before asleep). Players that completed NNT presented differences compared to the control group in sleep data. For instance, the lowest HR (*p* < 0.001), average HR (*p* < 0.001) and total awake time (*p* = 0.04) were significantly reduced in the NNT group. On the contrary, the control group presented greater values than the NNT group in the subjective Hooper Test, although only stress presented significant differences (Control 2.5 ± 1.2 vs. NNT cost or 3.2 ± 0.9; *p* = 0.01). Additionally, there were no significant differences in recovery parameters between weeks with one or two matches. In conclusion, the results suggest that players that underwent NNT tended to improve their sleep quality. Nevertheless, player’s values in the biochemical markers and wellness status remained similar in both groups. The fact that no significant differences were found between weeks with one or two matches could help basketball professionals to determine that a congested schedule does not seem to negatively alter recovery status.

**Clinical Trial Registration:** https://clinicaltrials.gov/ct2/show/NCT04939181?term=NCT04939181, NCT04939181

## Introduction

Basketball is one of the most popular team sports in the world, particularly in the United States and Europe, where high-level teams can play up to 100 official matches in a single season (Hulteen et al., 2017). More specifically, professional teams tend to accumulate between 2 and 3 matches per week during the season (Fox et al., 2020; Yang et al., 2021), meaning that players who accumulate significant playing time could struggle to reach optimal recovery after competition (Dellal et al., 2015; Calleja-González et al., 2016; Crowther et al., 2017). Thus, a detailed understanding of basketball players’ recovery during congested and non-congested schedules is critical to enhanced training prescription when the aim is to optimise the player’s in-game performance and health (Stojanovic and Ostojic, 2012; Weiss et al., 2017; Sansone et al., 2020).

To examine whether players are effectively coping with basketball competition, it is important to combine subjective and objective monitoring tools that can be implemented daily. Regarding subjective techniques, wellness questionnaires (e.g., Hooper test) of athlete readiness (Hooper et al., 1995; Haddad et al., 2013; Clemente et al., 2019) can provide useful data about a player’s perceptions regarding their recovery. For instance, the Hooper test is a valid and reliable (Laux et al., 2015) tool that analyses the player’s responses in four different parameters, namely sleep, stress, fatigue, and muscle soreness (Saw et al., 2016; Heidari et al., 2019). The sum of the different results yields a global index that has already been used in basketball (Clemente et al., 2019; García et al., 2022).

In addition to subjective techniques, common biochemical parameters such as testosterone, cortisol and their ratio, and sleep data from validated devices (e.g., “Oura” ring) are easy-to-use and widely applied tools that could help to complement wellness questionnaires and objectivize the decision-making process during training and competition.

Other authors have validated the usefulness of biomarkers, such as testosterone, cortisol and free testosterone to cortisol ratio, in describing the flexibility of the Autonomic Nervous System (ANS) to activate the sympathetic system at the beginning of exercise and, contrarily, the predominance of parasympathetic system when the activity ceases, showing the athlete’s response and their capacity to wind down and provide an efficient recovery process (Schelling et al., 2015; Lee et al., 2017; Greenham et al., 2018). Some researchers have demonstrated the relationship of cortisol and testosterone hormone levels and their testosterone to cortisol ratio for the purpose of describing sympathetic and parasympathetic processes related to training load during the competition season (Hoffman et al., 1999; Martínez et al., 2010; Ponce-González et al., 2015; Schelling et al., 2015; Arruda et al., 2017; Moreira et al., 2018; Kamarauskas and Conte, 2022). Furthermore, cardiopulmonary variables such as heart rate variability (HRV), resting heart rate (HRrest) and respiratory rate (RR) might be a reliable and complementary indicator to describe the training or competition load adaptation process.

Recently, several non-invasive devices have emerged to monitor these specific cardiopulmonary outcomes accompanied by sleep quality and quantity assessment providing additional and objective information about individual athletes’ responses (Schmitt et al., 2015; Bellenger et al., 2016).

The use of non-invasive neuromodulation techniques and devices are beginning to be investigated in sport to explore their effects on recovery and to study their contribution to performance and motor skills. For instance, Prismatic adaptation (Bonaventura et al., 2020) and stroboscopic training (Appelbaum et al., 2011) are based on visual activities combined with motor requirements of some sports technique to neuromodulate cortical areas. However, within the field of non-invasive neuromodulation treatments, a new technique called NESA (applied superficial neuromodulation) makes it possible to modulate changes in aspects related to the autonomic nervous system. In this case, it is a passive electrical neuromodulation, without combining motor activities or visual stimulation. Instead of cortical activity. The NESA neuromodulation treatment (NNT) is based on the application of microcurrents during an estimated treatment time to enhance the recovery by the stimulation of the autonomic nerve systems. Furthermore, the NNT is non-invasive, time-efficient, and readily transportable monitoring tool. The technology may be a useful and effective tool in high-level players to optimize recovery and content with exercise stressors (Medina-Ramírez et al., 2021).

Therefore, the objective of this study was to describe and compare recovery parameters (e.g. Hooper test, biochemical and sleep variables) 2 days after the match played at the weekend between male professional basketball players that underwent the NESA non-invasive neuromodulation treatment (NNT) over different types of in-season microcycles (congested and non-congested schedules). The authors of this study hypothesized that NNT may positively contribute to autonomic system modulation, indeed, increasing sleep quantity and quality which results in better recovery. Furthermore, recovery parameters were expected to decrease during congested basketball schedules.

## Materials and methods

### Subjects

The 12 professional male basketball players (mean ± SD, age: 20.6 ± 2.7 yr; height: 197.8 ± 11.7 cm; and body mass: 89.0 ± 21.2 kg) that participated in this study belonged to a reserve squad of a Spanish Euroleague team and competed in the LEB Plata (Spanish third division). The team usually practiced between three and 5 days a week between 10 a.m. and 13 h pm (1 h of strength and conditioning and 2 h of basketball training). Besides, the team played between one and two games per week, depending on the schedule. During the study, players from both groups continue to apply their usual recovery strategies, like cold-water immersion, foam rolling and cryotherapy. The new variation was the introduction of NNT protocol (see methodology section).

## Ethics

Before the research commenced, all the players were informed about the procedures and agreed to participate by providing their written consent. In addition, the Clinical Research Ethics Committee of the Catalan Sports Council (Government of Catalonia), with number 006/CEICGC/2021, approved the study’s experimental procedures (registration number NCT0493918). As it was a randomized controlled trial, the recommendations provided for by the Consolidated Standards of Reporting Trials Statement were followed.

### Study design

**FIGURE 1:**
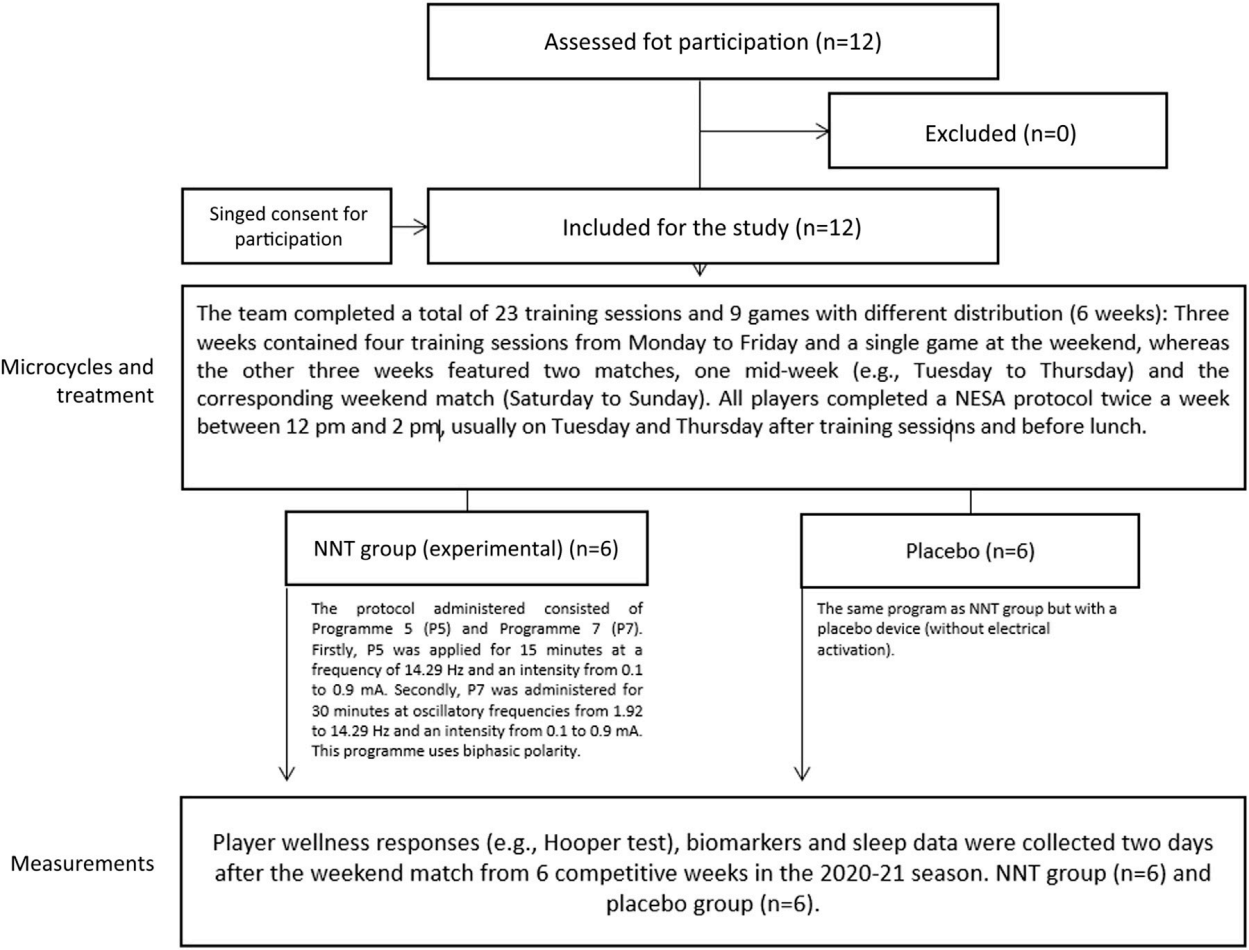
Flowchart of the study design.

A randomized clinical trial was used to examine the differences in recovery status between basketball players that completed a NNT and a placebo group during different types of in-season microcylcles. Player wellness responses (e.g., Hooper test), biomarkers and sleep data were collected 2 days after the weekend match from 6 competitive weeks in the 2020–21 season. The team completed a total of 23 training sessions and 9 games with different distribution: Three weeks contained four training sessions from Monday to Friday and a single game at the weekend, whereas the other 3 weeks featured two matches, one mid-week (e.g., Tuesday to Thursday) and the corresponding weekend match (Saturday to Sunday). Inclusion criteria was that players had to remain completely healthy during the intervention and only competed with their team (without playing with lower or higher-level teams from the same club). Furthermore, players that were injured during the game or did not play a minimum total time of 5 minutes were excluded from the analysis (Vázquez-Guerrero et al., 2019), any players were finally excluded. (Figure 1).

### Methodology

#### Procedures

The team played one or two games a week after a standard 45-min warm-up consisting of dynamic stretching, specific mobility exercises and individual basketball-specific skills such as shooting, passing and dribbling. Match-play was conducted according to official international basketball association (FIBA) rules. Additionally, the team followed a team sports-specific methodology called “structured training”, developed by FC Barcelona for the purpose of preparing athletes to compete in team sports (E Pons et al., 2020) and based on two types of training: coadjuvant (general off-court training. e.g., split squat and single-arm press) and optimization (sport-specific, on-court training. e.g., small-sided games and 5 on 5 scrimmage) (Gómez et al., 2019; Pons et al., 2020). In each microcycle, the team usually rested the day after a match.

#### NNT protocol

A randomized controlled trial was conducted to divide players through a randomization software into two groups: the placebo treatment group and the NNT group. All players completed a NESA protocol twice a week between 12 p.m. and 2 p.m., usually on Tuesday and Thursday after training sessions and before lunch. The assignment process was conducted blind by the supporting investigator in the team indicated for this study. To know if there were differences in playing times between groups (NESA vs. placebo) or factors (one match a week vs. two matches a week), we used a bootrsapped 2x2 permutation ANOVA to find differences in playing time variable of match days. The results were that there weren’t significant differences between groups or factors and that all players had the same amount of playing time in court.

NESA technique is a coordinated NNT through 24 electrodes, modulating the autonomic nervous system through ultralow-frequency electrical signals. The action on the different areas of the body is through the circulating bioelectricity current. The technology is minimally invasive; as a surface-based application. The characteristics of the current are an emission of low-frequency pulses oscillating from 1.3 Hz to 14.28 Hz (depending on the program), pulse emission at an intensity of 0.1–0.9 milliamps with a potential difference of ±3 V, with coordination between 24 electrodes (6 electrodes per limb, situated in both wrist and both ankles) stimulated simultaneously. The effect is not in a local muscle or nerve area activation, the effect is systemic due to the 24 electrode and the microcurrent produced by the ultra-low electrical parameters. The NNT protocol administered consisted of Program 5 (P5) and Program 7 (P7). Firstly, P5 was applied for 15 min at a frequency of 14.29 Hz and an oscillatory intensity from 0.1 to 0.9 mA. Secondly, P7 was administered for 30 min at oscillatory frequencies from 1.92 to 14.29 Hz and an intensity from 0.1 to 0.9 mA. This program uses biphasic polarity. The patient did not perceive a physical sensation of current due to the parameters used.

Even although all 12 players were connected to NESA units for the 45-min intervention, only half of the devices were adequately configured, whereas the other half were used only by the placebo group. While connected, all players rested in a seated or lying position on mats.

#### Recovery measures

A total of three different screening techniques were used to examine the player’s recovery status on MD+2:

##### Wellness status

Player subjective wellness was assessed using the Hooper questionnaire (Hooper et al., 1995), in which the players rated four categories (stress, fatigue, sleep, and delayed-onset muscle soreness) from 1 (very good) to 7 (very bad) approximately 30 min before each basketball session. Players became familiar with both numerical instruments during the pre- and in-season, beginning 6 months before the start of the experiment.

##### Biochemical parameters

The analysis was conducted using salivary samples. The samples were collected using DRG Instruments GmbH, Salivary Testosterone by ELISA and DRG Cortisol ELISA for free testosterone and cortisol, respectively. Both essays were performed in solid-phase enzyme-linked immunosorbent assay (ELISA) based on the competitive binding principle using ELISA Triturus analysers (Grifols, Spain). The testosterone:cortisol ratio was estimated after the testosterone and cortisol values had been divided. Cortisol is known as a hormone responsible of the athlete’s physiological responses at the beginning of physical activity, specifically where the Autonomic Nervous System (ANS) receives the information that the homeostasis of the body is modified from the baseline situation at the beginning of exercise and the sympathetic system is activated to supply the activity needed (Martínez et al., 2010; Fu and Levine, 2013; Schelling et al., 2015)). In contrast to cortisol, testosterone is related to anabolic processes, with parasympathetic system activation playing a relevant role in the recovery phase after physical stress resulting from exercise (Martínez et al., 2010; Schelling et al., 2015). Finally, the testosterone:cortisol ratio is useful in defining the balance between catabolic (cortisol) and anabolic (testosterone) processes and in describing the tendency of a player’s stress-recovery periods throughout the microcycle or season (Filaire et al., 2001; Elloumi et al., 2003; Schelling et al., 2015).

##### Sleep parameters

All the players were issued with the Oura ring, a commercially available wearable sleep and activity tracker (Oura Health, Oulu, Finland), which they wore on the finger of their choice around the clock (except for training sessions and games). At the beginning of the study, each participant downloaded the Oura application from their mobile phones and created an Oura account. The participants were asked to open the application every morning to upload the data from the ring to the application. Uploaded data were automatically transferred *via* an Internet connection to the study database in the Oura cloud service. The Oura ring measures sleep, and recovery variables based on resting heart rate (HRrest), heart rate variability (Rmssd) and motion using plethysmography and an accelerometer. The Oura ring classifies sleep epochs into four categories of sleep: wake, light, deep and REM sleep, previous researchers concluded a 57% accuracy in sleep phase classification, which was the main reason why we focused only on validated sleep variables such as ‘awake’ and ‘sleep’ in the current study. This study chose the following variables: 1) lowest night-time HR (bpm); 2) average night-time HR (bpm); 3) heart rate variability (Rmssd, ms); 4) sleep duration (h); 5) awake time (k); and 6) onset latency (h) to examine player’s quality of sleep (Altini and Kinnunen, 2021).

### Statistics

All the statistical analyses were conducted with RStudio version 1.3.1093 (RStudio, Inc.). Descriptive results were reported as mean ± standard deviation. Most of the variables studied failed all the tests for homogeneity of variance (Levene’s test) and normality (Shapiro–Wilk test). Due to this kind of data distribution, a bootstrapped 2 × 2 permutation ANOVA was conducted to identify significant differences in all the studied variables. The two independent variables used in the 2 × 2 model were the kind of group to which the players belonged (Placebo vs. Treatment) and the kind of week analyzed (one match a week vs. two matches a week). All the reported *p*-values were the likelihoods of the absolute effect sizes being observed if the null hypothesis of zero difference was true (Plonsky, 2015). We added the eta-squared value (η^2^) in order to describe the effect size. Finally, to permit an exhaustive discussion, we added the descriptive results from the familiarisation week data only from the variables with significant differences.

## Results

**FIGURE 2:**
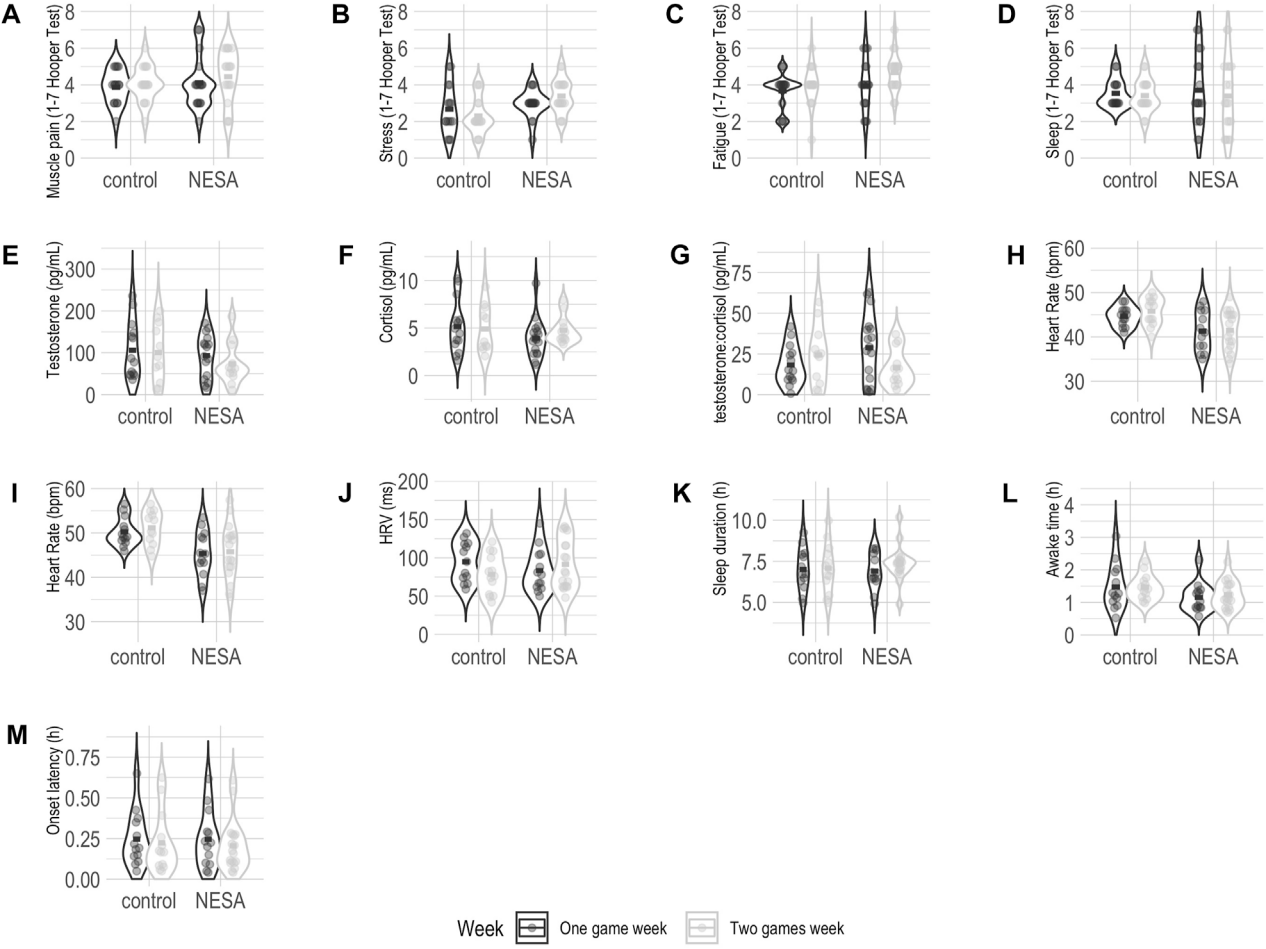
Distribution of all variables studied by group and week. **(A)** Hooper Delayed onset muscle soreness questionnaire. **(B)** Hooper Stress Questionnaire. **(C)** Hooper Fatigue questionnaire. **(D)** Hooper Sleep questionnaire. **(E)** Testosterone. **(F)** Cortisol. **(G)** Testosterone:Cortisol ratio. **(H)** Lowest night-time HR. **(I)** Average night-time HR. **(J)** Heart rate variability. **(K)** Sleep duration. **(L)** Awake time. **(M)** Onset latency.

The descriptive results of all the variables and the results from the 2 × 2 permutation ANOVA are presented in Table 1, and the visual representation of the results in Figure 2. Significant differences were only found between groups (Placebo vs. NNT) in the stress reported in the Hooper questionnaire (F Iterations = 294, p = 0.005, η^2^ = 0.11), in the lowest night-time HR (F Iterations = 5000, p < 0.001, η^2^ = 0.21), the average night-time HR (F Iterations = 5000, p < 0.001, η^2^ = 0.24) and night-time awake time (F Iterations = 3418, p = 0.02, η^2^ = 0.07). The main effects and interaction effects can be checked in Table 2.

**TABLE 1:** Descriptive and two-way ANOVA results of the MD+2 training session after the weekend match.

	Control group						NNT group						*p* (η^2^)		
	One game week			Two games week			One game week			Two games week			Group	Week	Group X week
**Hooper test**															
Muscle pain (1–7 AU)	3.87	±	0.92	4.06	±	0.97	4.12	±	1.41	4.44	±	1.42	0.3 (0.01)	0.4 (0.01)	0.8 (<0.01)
Stress (1–7 AU)*	2.67	±	1.35	2.29	±	0.99	2.94	±	0.75	3.39	±	0.92	0.01 (0.11)	0.9 (<0.01)	0.1 (0.03)
Fatigue (1–7 AU)	3.67	±	0.98	3.94	±	1.09	3.94	±	1.34	4.67	±	1.03	0.1 (0.04)	0.1 (0.04)	0.4 (0.01)
Sleep (1–7 AU)	3.53	±	0.74	3.41	±	0.87	3.71	±	1.96	3.39	±	1.97	0.9 (<0.01)	0.6 (<0.01)	0.8 (<0.01)
**Biomarkers**															
Testosterone (pg/ml)	106	±	67.3	100	±	69.1	93.3	±	51.3	73.6	±	49.3	0.3 (0.02)	0.4 (0.01)	0.7 (<0.01)
Cortisol (pg/ml)	6.38	±	5.48	4.91	±	2.43	3.91	±	2.03	4.77	±	1.45	0.1 (0.05)	0.7 (<0.01)	0.2 (0.03)
Testosterone:cortisol (pg/ml)	22.4	±	20.2	24.3	±	18.2	28.9	±	20.6	16.4	±	11.5	0.9 (<0.01)	0.3 (0.02)	0.2 (0.03)
**Oura data**															
Lowest HR (bpm)*	44.6	±	2.22	45.8	±	3.11	41.3	±	4.55	41.5	±	4.41	< 0.001 (0.21)	0.5 (<0.01)	0.6 (<0.01)
Average HR (bpm)*	50.3	±	3.22	52.0	±	4.68	45.4	±	5.11	45.8	±	6.07	< 0.001 (0.24)	0.4 (<0.01)	0.6 (<0.01)
HRV (ms)	94.8	±	26.0	78.2	±	25.9	83.3	±	28.1	91.4	±	32.2	0.8 (<0.01)	0.6 (<0.01)	0.1 (0.04)
Sleep duration (h)	7.01	±	1.28	7.08	±	1.48	6.91	±	1.10	7.38	±	1.06	0.7 (<0.01)	0.4 (0.01)	0.5 (<0.01)
Awake time (h)*	1.47	±	0.70	1.47	±	0.39	1.15	±	0.45	1.23	±	0.42	0.04 (0.07)	0.8 (<0.01)	0.7 (<0.01)
Onset latency (h)	0.25	±	0.17	0.22	±	0.19	0.25	±	0.17	0.21	±	0.16	0.8 (<0.01)	0.5 (<0.01)	0.9 (<0.01)

Note. * Significant differences between groups. † Significant differences between weeks. ‡ Significant differences interaction effect. NNT, is NESA, neuromodulation treatment; HR, is heart rate; HRV, is heart rate variability.

**TABLE 2:** Main effects and interaction effects of the two–way ANOVA results in all studied variables.

	Main effects group		Main effects week		Interaction Group:Week	
	F	Permutation p	F	Permutation p	F	Permutation p
**Hooper test**						
Muscle pain (1–7 AU)	1.145	0.286	0.761	0.379	0.051	0.816
Stress (1–7 AU)	7.684	0.001	0.023	0.874	2.757	0.098
Fatigue (1–7 AU)	3.315	0.068	3.131	0.072	0.674	0.417
Sleep (1–7 AU)	0.039	0.861	0.342	0.551	0.067	0.786
**Biomarkers**						
Testosterone (pg/ml)	1.438	0.272	0.587	0.440	0.184	0.665
Cortisol (pg/ml)	2.029	0.147	0.114	0.744	1.635	0.210
Testosterone:cortisol (pg/ml)	0.020	0.883	1.093	0.302	2.032	0.153
**Oura data**						
HR lowest (bpm)*	14.081	0.001	0.444	0.520	0.226	0.636
HR average (bpm)*	17.162	0.001	0.617	0.433	0.231	0.623
HRV (ms)	0.012	0.814	0.307	0.583	2.619	0.105
Sleep duration (h)	0.094	0.737	0.678	0.424	0.370	0.540
Awake time (h)*	4.381	0.043	0.081	0.777	0.111	0.747
Onset latency (h)	0.039	0.841	0.047	0.503	0.034	0.858

The descriptive results, grouped only by week analyzed or by treatment group, are presented in Table 3. Finally, the results from the familiarization period only with the variables with significant differences are presented in Table 4.

**TABLE 3:** Descriptive data of the MD+2 training session after the weekend match by week and by group.

	Placebo group			NNT group			One game week			Two games week		
**Hooper test**												
Muscle pain (1–7 AU)	3.97	±	0.93	4.29	±	1.41	4.00	±	1.19	4.26	±	1.22
Stress (1–7 AU)	2.47	±	1.16	3.17	±	0.86	2.81	±	1.06	2.86	±	1.09
Fatigue (1–7 AU)	3.81	±	1.03	4.31	±	1.23	3.81	±	1.18	4.31	±	1.11
Sleep (1–7 AU)	3.47	±	0.80	3.54	±	1.95	3.63	±	1.50	3.40	±	1.52
**Biomarkers**												
Testosterone (pg/ml)	104	±	66.7	84.9	±	50.5	99.3	±	58.6	86.5	±	59.7
Cortisol (pg/ml)	5.76	±	4.44	4.27	±	1.83	5.07	±	4.15	4.84	±	1.93
Testosterone:cortisol (pg/ml)	23.2	±	19.0	23.7	±	18.3	25.9	±	20.4	20.2	±	15.3
**Oura data**												
Lowest HR (bpm)	45.2	±	2.71	41.4	±	4.40	43.0	±	3.89	43.3	±	4.41
Average HR (bpm)	51.1	±	4.03	45.6	±	5.60	47.8	±	4.87	48.4	±	6.26
HRV (ms)	86.5	±	26.8	88.0	±	30.4	89.1	±	27.2	85.9	±	30.0
Sleep duration (h)	7.04	±	1.35	7.18	±	1.08	6.96	±	1.17	7.25	±	1.24
Awake time (h)	1.47	±	0.55	1.20	±	0.43	1.31	±	0.60	1.33	±	0.42
Onset latency (h)	0.23	±	0.17	0.22	±	0.16	0.25	±	0.17	0.21	±	0.17

Note. NNT, is NESA, neuromodulation treatment; HR, is heart rate; HRV, is heart rate variability.

**TABLE 4:** Descriptive results of the familiarisation period of the variables with significant differences in the 2 × 2 ANOVA.

	Placebo group			NNT group		
Stress (1–7 AU)	2.83	±	1.07	2.77	±	0.63
Lowest HR (bpm)	47.2	±	2.85	43.3	±	4.24
Average HR (bpm)	53.7	±	3.67	48.3	±	5.62
Awake time (h)	1.38	±	0.42	1.37	±	0.53

Note. NNT, is NESA, neuromodulation treatment; HR, is heart rate; HRV, is heart rate variability.

## Discussion

The aim of this study was to describe and compare the recovery status between basketball players that underwent the NNT and a placebo group in weeks with one and two matches. The primary finding of this study is that players that completed two NNT sessions a week presented significantly reduced values in sleep parameters (lowest HR, average HR, and total awake time). Additionally, there were no significant differences in recovery variables between weeks with one or two matches, which might suggest that a congested schedule in basketball does not seem to negatively alter recovery status.

Even although there are no published studies demonstrating the effect of NNT on professional basketball players’ recovery status, this investigation found that some sleep variables were significantly greater in the intervention group compared to the placebo group. Specifically, players that underwent the NNT presented better values in the lowest HR, average HR and total awake time and during the 6-week intervention. Nevertheless, the results should be interpreted with caution since differences between groups in sleep parameters already existed before the experiment (Table 3). In addition to sleep data, this study did not find significant differences between the NNT and placebo group in any of the three biomarkers examined (cortisol, testosterone, and their ratio). However, monitoring cortisol could be interesting since professional basketball players presented significantly higher values during official competition compared to simulated matches (Moreira et al., 2012). Moreover, the fact that sleep restriction is strongly associated with higher cortisol levels the day after (Leproult et al., 1997; Oginska et al., 2010) might strengthen the idea that sleep quality is an important variable to monitor in professional players to optimize recovery.

As with previous research (García et al., 2022) using the Hooper test to assess player responses to basketball training and competition, this study only detected significant differences in the stress variable. In this sense, García et al. (2022) concluded that basketball players that reported stable stress values during the previous week achieved significantly better match performances compared to players with high variability in the same variable. Similarly, the Hooper test was also useful in detecting differences between basketball-specific playing positions: backcourt players managed to maximize their performance in comparison to frontcourt players when fatigue levels were stable during the macrocycle. Although this study did not examine the stability and variability of the Hooper test values, the fact that stress presented significant differences might demonstrate that it could be important to monitor this parameter to understand player performance better.

Regarding competition congestion, our study did not find any significant differences in recovery parameters. However, the players reported a generally non-significant increase in wellness values (stress, fatigue, and DOMS) and a decrease in HRV in weeks with two matches. Similarly, Clemente et al. (2019) also concluded that weeks with two matches presented moderately greater fatigue (*p* = 0.47; d = 1.40, moderate effect) and lower sleep quality (*p* = 0.42; d = 1.32, moderate effect) than weeks with one match. Despite these results, basketball players can apparently cope with different types of congested match schedules after the failure to detect significant differences in training load, readiness, and recovery status (Clemente et al., 2019; Pino-Ortega et al., 2019; Fox et al., 2020; Lukonaitiene et al., 2021).

The fact that a non-invasive neuromodulation device in the form of NESA technology (NNT) is available to a professional team enables it to be integrated into its daily training routine, and it can be used as a convenient, non-invasive treatment with no side effects. Optimizing the athlete and the autonomic nerve system can lead to improved future performance and improved sleep. In recent years, elite athletes’ sleep quality has been seen to be impacted by their schedules and the demands placed upon them, meaning that increasing this improvement could lead, in turn, to optimize performance.

Our research is the first study in the world to be conducted using NESA non-invasive treatment in the elite sports setting. It is also the first placebo-compared clinical trial in this area of neuromodulation. However, here are some limitations of this study that should be considered: 1) this study includes a small sample size, as the data were collected from a single basketball team, which does not renders it impossible to mainstream our findings to other types of competitions and basketball populations; 2) recovery variables were only collected 2 days after the weekend competition; and 3) only player response variables (e.g., Hooper test, biochemical and sleep data) were considered for the assessment of recovery. Therefore, future research should examine a broader variety of recovery parameters (including physical and physiological parameters) over different time periods (e.g. immediately after the match and after 24 and 72 h) in different populations (e.g., young, semi-professional, female players) participating in different types of competition (e.g., congested tournaments, pre-season, and play-off matches) to obtain a better understanding of the relationship between the conditional structure and basketball performance.

## Conclusion

In conclusion, the results suggest that players who underwent the NNT tended to improve their sleep quality even although their biochemical markers and wellness status remained similar to the placebo group. Furthermore, the fact that no significant differences were found between weeks with one or two matches could help basketball professionals to determine that a congested schedule does not seem to negatively affect recovery status.

## Key points

• The Hooper test could become a valid and reliable subjective strategy to assess player responses to basketball training and competition.
• The NESA non-invasive neuromodulation electrotherapy could help sport players to better recover after training and competition.
• Basketball players perfectly cope with a congested schedule consisting in two matches a week without showing significant alterations in recovery parameters.

## Data Availability

The raw data supporting the conclusion of this article will be made available by the authors, without undue reservation.
